# MHD Casson fluid flow with Navier’s and second order slip due to a perforated stretching or shrinking sheet

**DOI:** 10.1371/journal.pone.0276870

**Published:** 2022-11-04

**Authors:** Jitender Singh, A. B. Vishalakshi, U. S. Mahabaleshwar, Gabriella Bognar

**Affiliations:** 1 Department of Mathematics/Guru Nanak Dev University, Amritsar, India; 2 Department of Mathematics/ Davangere University Shivagangotri, Davangere, India; 3 Institute of Machine and Product Design/ University of Miskolc, Miskolc, Hungary; NUST: National University of Sciences and Technology, PAKISTAN

## Abstract

The present work discusses the laminar boundary layer flow of an electrically conducting Casson fluid due to a horizontal perforated sheet undergoing linear shrinking/stretching with mass transpiration. Navier’s slip and second-order slip conditions are also imposed on the flow. The system is subjected to a transverse magnetic field. The non-Newtonian flow under consideration obeys the rheological equation of state due to the Casson model. The PDEs governing the bounder layer flow is reduced to a nonlinear boundary value problem in ODEs by utilizing appropriate similarity transformations and are expressed analytically. The similarity solution is found to be a function of the Casson parameter, magnetic parameter, mass suction/injection parameter, and the first/second-order slip parameters. Such a solution is either unique, or dual solutions exist in a region defined by the mass transfer induced slip parameter. The results of the present work are found to be an increase of the magnetic effects resulting in expansion of the unique solution region and contraction of the dual solution region for the flow due to the induced Lorentz force. In the unique solution region, an increase in magnitudes of mass suction induced slip and the first/second-order slip parameters result in a reduction of the wall shear stress in the shrinking sheet, while the wall shear stress with mass suction increases with the Casson and the magnetic effects. Similar results exist for the stretching sheet case with mass suction. However, only unique similarity solutions exist only for the case of stretching sheets with mass injection. The current work is a generalization of the classical works of Crane (1970) and Pavlov (1974) for a stretching sheet. Mass suction/injection induced slip enhances and achieves a dominant flow driven by reversing the flow direction of the moving sheet, which allows an adjacent flow against the sheet. The findings have possible industrial applications in fluid-based systems including stretchable/shrinkable things, automated cooling systems, power generation, microelectronics, and present new results to the problem.

## 1 Introduction

Mathematical modeling of nonlinear physical phenomena occurring in biology, physical sciences, pharmaceutical, and engineering sciences often results in a system of highly nonlinear differential equations. Applications of stretching sheet dynamics generally occur in polymer extrusion processes involving the cooling of continuous strips extruded from a dye through a stagnant fluid. Distinctive sorts of non-Newtonian liquids as well as different modeling approaches have been utilized in the past to depict and clarify the conduct of non-Newtonian flow in these physical situations.

The Casson fluid which is a standout amongst the most critical non-Newtonian rheological models is a plastic fluid that displays shear subordinate attributes and additional yield stress. The Casson fluid flow occurs when the shear stress exceeds the yield stress. The Casson model was created for liquids containing bar-like solids and is frequently connected to model bloodstream and other practical applications such as modern handling of liquid chocolate and related foodstuff. The flow incited by stretching the boundary in the polymer removal, drawing of copper wires, constant extending of plastic films and recreated strands, hot moving glass fibers, metal ejection, and metal turning is a segment of the situations where the phenomenon of a stretching boundary develops. A day to day increased use of non-Newtonian fluids in industrial applications has increased the interest of researchers in theoretical and experimental investigations on the flow characteristics of such complex fluids. As far back as the spearheading works of Blasius [1] and Sakiadis [2, 3], Crane [4]obtained an analytical solution of the boundary layer equations for the flow due to stretching of a plastic sheet in the polymer industry. Recently, Bhattacharya et al. [5] obtained closed-form solutions for the steady boundary layer flow of a Casson fluid over a permeable stretching/shrinking sheet. Their analysis reveals that the solution is unique for the stretching sheet case. On the other hand, depending upon the Casson parameter, the solution for the shrinking sheet case may not exist at all, or there may be a unique solution, or multiple solutions may exist. Hussanan et al. [6] have obtained similarity solutions in terms of hypergeometric functions for the boundary layer flow of a steady viscoelastic Casson fluid flow past a stretching surface under mass transpiration and viscous dissipation. Bhatti et al. [7] studied the mass transfer process by considering Jeffrey fluid model, in this method he uses the robust computational approach to examine the mass transfer process. Chu et al. [8] worked on the impact of Cattaneo-Christov double diffusion and radiative heat flux on the flow of Maxwell liquid due to stretched nanomaterial surface. Wang et al. [9] examined the non-Newtonian fluid flow in the presence of heat generation/absorption and radiative heat flux. Khan et al. [10] concluded the outcomes for chemically reactive aspects in the flow of tangent hyperbolic material. Hayat et al. [11, 12] numerically investigated the nonlinear radiative flow in a convective cylinder. And also, they demonstrated the squeezing flow of the second grade liquid subject to non-Fourier heat flux and heat generation/absorption. Qayyum et al. [13] investigated the comparison of five nanoparticles with the viscous flow in the presence of slip and rotating disc. Safdar et al. [14] worked on the unsteady flow of a liquid film due to stretching sheets using file point symmetries. Aziz and Mahomed [15] studied the theoretical methods for non-Newtonian fluid flow and also its applications. Paliathanasis [16] worked on Lie symmetries using rotating shallow water. Mekheimer et al. [17] investigated the lie point symmetries for an electrically conducting Jeffrey fluid.

The steady, laminar MHD boundary layer flows driven by moving boundaries are widely studied flow problems [18]. Pavlov [19] investigated an MHD laminar boundary layer flow of an electrically conducting liquid due to a stretching sheet in the presence of a transverse magnetic field under the assumption of very small magnetic Reynolds number. Chakrabarti and Gupta [20] extended the work of Crane [4] by including the effect of a transverse magnetic field to the MHD flow over a stretching sheet. The changelessness of the accurate results was researched by Takhar et al. [21]. Fang and Zhang [22] obtained closed form similarity solutions for the MHD viscous flow of a Newtonian fluid over a shrinking sheet under mass suction/injection and found multiple solution branches depending upon the applied magnetic field. In fact the studies pursued by Bhattacharya et al. [23] on the steady boundary layer flow of a Casson fluid over a permeable stretching/shrinking sheet under MHD conditions establish that the effect of increasing the applied magnetic field results in widening of the parameter space of unique solutions. So, the problem of MHD boundary layer flows over a stretching/shrinking sheet under various physical conditions has become a paradigm [6, 24–30]. Zhang et al. [31] worked on hybrid nanofluid flow in the presence of an induced magnetic field, in this study the flow passes towards the elastic surface having tantalum and nickel nanoparticles. Nazeer et al. [32] studied the MHD electro-osmotically flow of third grade fluid theoretically in the presence of a microchannel.

**Fig 1:**
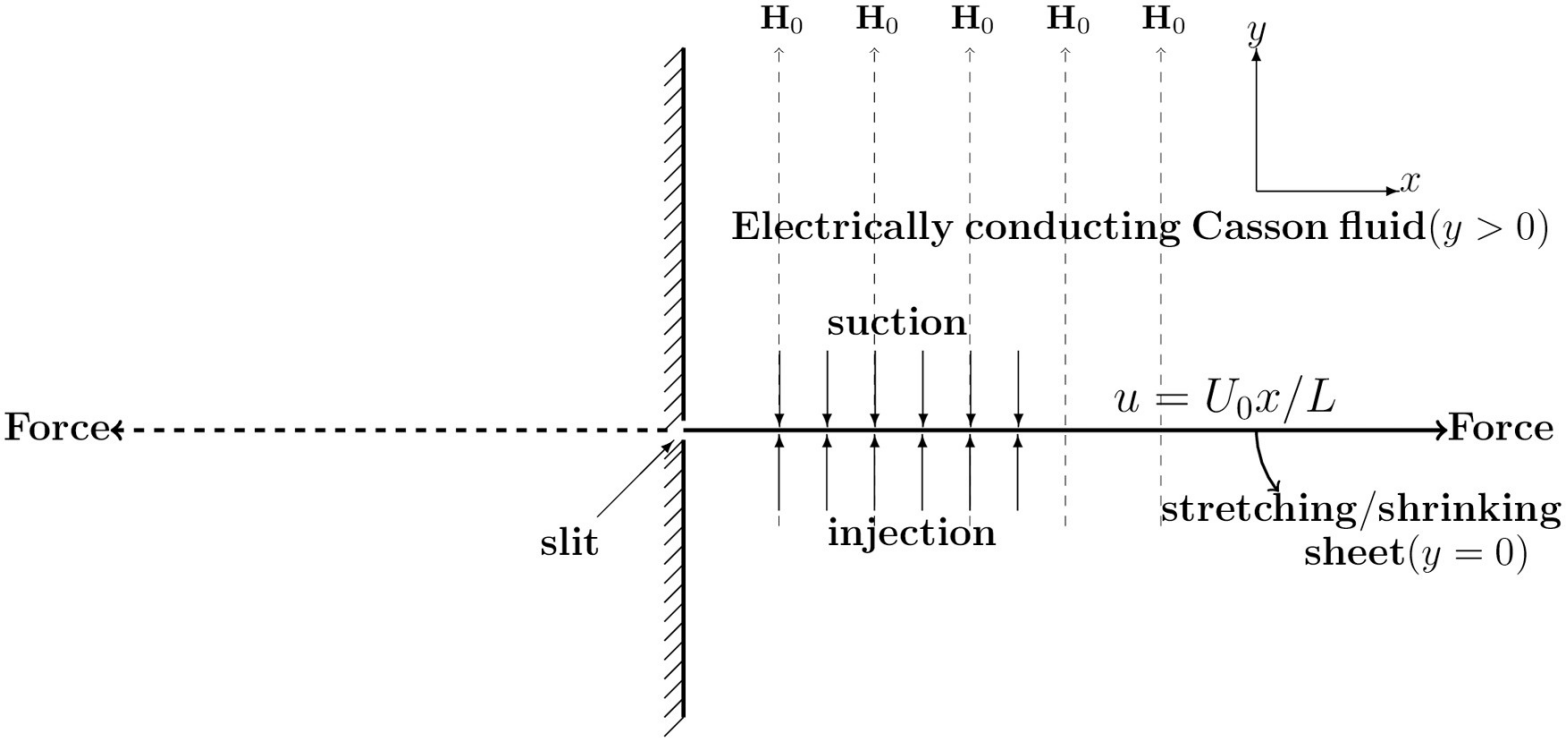
Physical model when the system is subjected to a vertical magnetic field H_0_.

As pointed out by Vleggaar [33] in a polymer processing application including spinning of filaments without blowing, the boundary layer happens generally over a small length of the zone of about 0.0-0.5 meters from the dye which might be taken as the starting point of Fig 1. In fact, this is the region beyond which much of the stretching takes place. In such a progression, the preliminary velocity is low (about 0.3 m/s) but not very low enough always to assume the linear stretching. Thus an excellent estimate of the velocity of the sheet is *u* = *U*_0_(*x*/*L*)^*n*^ (at any rate for the first 10-60 cm of the whirling region) where L is the characteristic length for measuring horizontal distance and n is the stretching sheet parameter to model the nonlinearity.

Recently, Mahabaleshwar et al. [34] have investigated the laminar boundary layer Casson fluid flow past a stretching/shrinking sheet under MHD conditions and found the closed-form analytic solutions for the flow field. The dual solutions have been reported for the case of a shrinking sheet. Their analytic and numerical results indicate the dependence of the flow and the wall shear stress on the Casson effects, the mass suction and injection, and the MHD environment.

The present research is focused on the MHD flow of a Casson fluid due to a perforated sheet undergoing linear stretching/shrinking with mass transfer and with consideration of Navier’s and second order slip velocity conditions. Thus, the problem investigated in this paper is a generalization (See Table 1 for a quick comparison of the present work with the past literature) of the classical work of Crane [4], Pavlov [19], and the recent works of Fang and Zhang [22], Fang et al. [35], Bhattacharya et al. [5, 23], Wu [36], and Mahabaleshwar et al. [34].

**Table 1:** Solutions deductible from present formulation. The symbol A refers to as any permissible value of the corresponding parameter.

Reference	*γ*	*M*	*γ*_1_	*γ*_2_	*V*_*c*_	Closed form solution
Crane(1970) [4]	∞	0	0	0	0	*f*(*η*) = 1−*e*^−*η*^
Pavlov(1974) [19]	∞	A	0	0	0	$f\left(\eta \right)=\frac{1}{\sqrt{1+M}}\left(1-e^{-\sqrt{1+M}\eta }\right)$
Fang and Zhang (2009) [22]	∞	A	0	0	A	$f\left(\eta \right)=V_{c}-\;\frac{1}{\alpha }\left(1-e^{-\alpha \eta }\right)$ , $\alpha =(V_{c}\pm \sqrt{V_{c}^{2}-4\left(1-M^{2}\right)})/2$
Fang et al.(2010) [35]	∞	0	A	A	A	$f\left(\eta \right)=V_{c}-\frac{1}{\alpha +\gamma_{1}\alpha^{2}-\gamma_{2}\alpha^{3}}\left(1-e^{-\alpha \eta }\right)$ , *α* satisfying (14), *d* = −1
Bhattacharya et al.(2013) [5]	A	0	0	0	A	$f\left(\eta \right)=V_{c}+\frac{1}{\alpha }\left(1-e^{-\alpha \eta }\right)$
						$\alpha =\frac{V_{c}+\sqrt{V_{c}^{2}+4\left(1+1/\gamma \right)\left(1+M\right)}}{2(1+1/\gamma )}$ (shrinking)
						$\alpha =-\frac{V_{c}\pm \sqrt{V_{c}^{2}-4\left(1+1/\gamma \right)}}{2(1+1/\gamma )}$ (stretching)
Bhattacharya et al.(2014) [23]	A	A	0	0	A	$f\left(\eta \right)=V_{c}+\frac{1}{\alpha }\left(1-e^{-\alpha \eta }\right)$
						$\alpha =\frac{V_{c}+\sqrt{V_{c}^{2}+4\left(1+1/\gamma \right)\left(1+M\right)}}{2(1+1/\gamma )}$ (shrinking)
						$\alpha =-\frac{V_{c}\pm \sqrt{V_{c}^{2}-4\left(1+1/\gamma \right)\left(1+M\right)}}{2(1+1/\gamma )}$ (stretching)
Lin Wu(2016) [36]	∞	0	A	A	A	*f*(*η*) = *α*+ (*V*_*c*_−*α*)(1−*e*^−*αη*^), *α* satisfying (14)
Singh et al.(2019) [43]	∞	A	0	0	A	$f\left(\eta \right)=V_{c}+\frac{1}{\alpha }\left(1-e^{-\alpha \eta }\right)$ , $\alpha =\frac{V_{c}+\sqrt{V_{c}^{2}+4\left(1+M\right)}}{2}$
(Linear stretching case)						
Mahabaleshwar et al.(2020) [34]	A	A	0	0	A	$f\left(\eta \right)=V_{c}+\frac{d}{\alpha }\left(1-e^{-\alpha \eta }\right)$
						$\alpha =\frac{V_{c}+\sqrt{V_{c}^{2}+4\left(1+1/\gamma \right)}}{2(1+1/\gamma )}$ (shrinking)
						$\alpha =-\frac{V_{c}\pm \sqrt{V_{c}^{2}-4\left(1+1/\gamma \right)}}{2(1+1/\gamma )}$ (stretching)
**Present work**	A	A	A	A	A	$f\left(\eta \right)=V_{c}+\frac{d}{\alpha (1+\gamma_{1}\alpha -\gamma_{2}\alpha^{2})}\left(1-e^{-\alpha \eta }\right)$ , *α* satisfying (14)

The novelty of the present work is to investigate the Casson fluid flow due to shrinking/stretching sheets in the presence of Navier’s slip and second order slip under the impact of the magnetic field. The PDEs of the governing problems are altered into ODEs by using similarity variables. The suction and injection parameter is also considered in the present work. The present problem is used in many industrial applications such as extrusion of polymer process, automated cooling systems, and entropy generation (see Zhao et al. [37], Hayat et al. [38] and Khan et al. [39]).

## 2 Mathematical model

Consider a laminar, steady boundary layer flow of an electrically conducting and incompressible Casson fluid that passes through a stretched perforated sheet (see Fig 1). Over the sheet, a laminar boundary layer flow is driven by a nonuniform motion of the sheet which is accelerating in the axial direction with *U*(*x*) = ±*U*_0_ *x*/*L* as the shrinking (negative sign) or stretching (positive sign) speed of the sheet, where *U*_0_ > 0 and *L* > 0 are the characteristic scales for measuring horizontal velocity and horizontal length respectively.

The system is subjected to a constant vertical magnetic field **H**_0_ = (0, *H*_0_). To study the dynamics of the flow induced by the stretching sheet in the plane *y* = 0, the conducting liquid is assumed in the half space *y* > 0. We examine Hartmann’s formulation of the MHD problem. The flow of the initially quiescent fluid is induced by pulling the sheet parallel to the sheet at both ends with equal and opposite force, resulting in a plate speed of *U*. The flow of otherwise quiescent fluid is only caused by the movement of the sheet. On the sheet, a constant suction rate of (0, −*V*_0_) is caused (see [18] Ch. 11, pp. 302). By convention, *V*_0_ > 0 is the suction, while *V*_0_ < 0 is the fluid injection at *y* = 0.

The rheological stress components for the flow of an incompressible Casson liquid is expressed as (see Nakamura and Sawada [40])

$$\begin{array}{c} \tau_{ij}=\{\begin{array}{c} (\mu_{B}+\frac{\tau_{y}}{\sqrt{2D}})(\frac{\partial u_{i}}{\partial x_{j}}+\frac{\partial u_{j}}{\partial x_{i}}),\;\text{if}\;D<D_{c};\\ (\mu_{B}+\frac{\tau_{y}}{\sqrt{2D_{c}}})(\frac{\partial u_{i}}{\partial x_{j}}+\frac{\partial u_{j}}{\partial x_{i}}),\;\text{if}\;D>D_{c}. \end{array} \end{array}$$ (1)

where *μ*_*B*_ is the active viscosity of the non-Newtonian liquid, *τ*_*y*_ is the yield stress of the liquid, *D* is the resultant component of deformation rate, *D*_*c*_ is the critical value based on the non-Newtonian model, and *u*_*i*_, *u*_*j*_ are the two fluid velocity components for *i*, *j* ∈ {1, 2}.

Let *Re* = *U*_0_ *L*/*ν* > 0 be the Reynolds number corresponding to the horizontal component of the flow. Observe that in the boundary layer theory, we have $\delta /L=O(Re^{-1/2})$ if *δ* denotes the thickness of the laminar boundary layer near the stretching sheet.

We take *δ* as the characteristic scale for measuring length along the vertical. We also use *U*_0_ *δ*/*L* as the scale for measuring vertical component of the fluid velocity. Using these considerations, the conservation of mass, zero pressure gradient laminar boundary layer equation for the Casson liquid as a result of perforated sheet undergoing stretching are given by

$$\begin{array}{ccc} \frac{\partial u}{\partial x}+\frac{\partial v}{\partial y} & = & 0;\;u\frac{\partial u}{\partial x}+v\frac{\partial u}{\partial y}=\nu (1+\frac{1}{\gamma })\frac{\partial^{2}u}{\partial y^{2}}-\frac{\sigma H_{0}^{2}}{\rho }u, \end{array}$$ (2)

where (*u*(*x*, *y*), *v*(*x*, *y*)) is the fluid velocity induced by the stretching/shrinking sheet; *ρ* is the fluid density, *ν* = *μ*_*B*_/*ρ* is the kinematic viscosity, and *σ* is the electrical conductivity of the fluid. The parameter *γ* is given by

$$\begin{array}{c} \gamma =\mu_{B}\frac{\sqrt{2D_{c}}}{\tau_{y}}, \end{array}$$ (3)

which is the ratio of the deformation and the yield stresses of the Casson fluid. The relevant boundary conditions for the present Casson model are given by [36, 41, 42]

$$\begin{array}{ccc} u(x,0) & = & U\left(x\right)d+(1+\frac{1}{\gamma })(A\frac{\partial u}{\partial y}\left(x,0\right)+B\frac{\partial^{2}u}{\partial y^{2}}\left(x,0\right)) \end{array}$$ (4)

$$\begin{array}{ccc} v(x,0) & = & -V_{0};\;\underset{y\to \infty }{\text{lim}}u\left(x,y\right)=0, \end{array}$$ (5)

where *U*(*x*) and *V*_0_ are as aforementioned, *d* is the parameter of proportional shearing at the boundary (*d* = 0 corresponds to the boundary at *y* = 0 and *d* ≠ 0 corresponds to proportionally sheared boundary). The constants *A* > 0 and *B* < 0 represent the first and second-order slip coefficients, respectively.

Let *ψ* be the stream function for the flow so that $\left(u,v\right)=\left(\frac{\partial \psi }{\partial y},-\frac{\partial \psi }{\partial x}\right)$. We define variable *η* and dimensionless stream function *f*(*η*) as

$$\begin{array}{c} \eta =\sqrt{Re{\left(\delta /L\right)}^{2}}y/\delta =\sqrt{Re}y/L;\;\psi =\nu \sqrt{Re}\left(x/L\right)f\left(\eta \right). \end{array}$$ (6)

Consequently, the fluid velocity components are given by

$$\begin{array}{c} u=U\left(x\right)f^{\prime }\left(\eta \right);\;v=-\left(V_{0}/V_{c}\right)f\left(\eta \right), \end{array}$$ (7)

where

$$\begin{array}{c} V_{c}=\left(V_{0}L/\nu \right)/\sqrt{Re} \end{array}$$ (8)

is the dimensionless suction/injection parameter. At the sheet surface, *V*_*c*_ > 0 corresponds to fluid suction and *V*_*c*_ < 0 corresponds to the fluid injection.

Substituting (6)-(7) in (2) and in (4)-(5), we have the following nonlinear third order two-point boundary value problem

$$\begin{array}{ccc} (1+\frac{1}{\gamma })f^{\prime \prime \prime }+ff^{\prime \prime }-f^{\prime 2}-Mf^{\prime } & = & 0, \end{array}$$ (9)

$$\begin{array}{ccc} f\left(0\right)=V_{c};\;\;f^{\prime }\left(0\right)-\left(d+\gamma_{1}f^{\prime \prime }\left(0\right)+\gamma_{2}f^{\prime \prime \prime }\left(0\right)\right) & = & 0;\;\;\underset{\eta \to \infty }{\text{lim}}f^{\prime }\left(\eta \right)=0, \end{array}$$ (10)

where 0 ≤ *η* < ∞ and the parameters $\gamma_{1}=\left(1+1/\gamma \right)A\sqrt{Re}/L\geq 0;\;\;\gamma_{2}=\left(1+1/\gamma \right)BRe/L\leq 0$ are the dimensionless forms of the modified first and second order slip parameters for the Casson model. We also have

$$\begin{array}{ccc} d & = & \pm (1+m_{1}), \end{array}$$ (11)

where positive and negative signs correspond to the stretching and shrinking sheet cases, respectively, and *m*_1_ is the dimensionless mass transfer induced slip parameter such that *m*_1_ *V*_*c*_ ≤ 0. The parameter *M* appearing in (6) is the magnetic parameter defined by

$$\begin{array}{c} M=Q/Re\geq 0, \end{array}$$ (12)

where $Q=\sigma L^{2}H_{0}^{2}/\left(\rho \nu \right)$ is Chandrasekhar number. The closed form solution of (9)-(10) is given by

$$\begin{array}{c} f\left(\eta \right)=V_{c}+\frac{d}{\alpha (1+\gamma_{1}\alpha -\gamma_{2}\alpha^{2})}\left(1-e^{-\alpha \eta }\right),\;\;\alpha >0, \end{array}$$ (13)

where *α* is a positive root of the following biquadratic polynomial equation

$$\begin{array}{c} a_{1}\alpha^{4}+a_{2}\alpha^{3}+a_{3}\alpha^{2}+a_{4}\alpha +a_{5}=0, \end{array}$$ (14)

wherein

$$\begin{array}{ccc} a_{1} & = & (1+\frac{1}{\gamma })\gamma_{2};\;\;a_{2}=-\{V_{c}\gamma_{2}+\gamma_{1}+\frac{\gamma_{1}}{\gamma }\};\;\;a_{3}=\{V_{c}\gamma_{1}-1-\frac{1}{\gamma }-M\gamma_{2}\}, \end{array}$$ (15)

$$\begin{array}{ccc} a_{4} & = & \{V_{c}+M\gamma_{1}\};\;\;a_{5}=M+d. \end{array}$$ (16)

Consequently, the four solutions *α* = *α*_1_, *α*_2_, *α*_3_, *α*_4_ of the biquadratic Eq (14) using the standard Ferrari’s method are given by

$$\begin{array}{ccc} \alpha_{1} & = & -\frac{a_{2}}{4a_{1}}+S+\frac{1}{2}\sqrt{-4S^{2}-2p-q/S};\;\;\alpha_{2}=-\frac{a_{2}}{4a_{1}}+S-\frac{1}{2}\sqrt{-4S^{2}-2p-q/S}, \end{array}$$ (17)

$$\begin{array}{ccc} \alpha_{3} & = & -\frac{a_{2}}{4a_{1}}-S+\frac{1}{2}\sqrt{-4S^{2}-2p+q/S};\;\;\alpha_{4}=-\frac{a_{2}}{4a_{1}}-S-\frac{1}{2}\sqrt{-4S^{2}-2p+q/S}, \end{array}$$ (18)

where

$$\begin{array}{ccc} p & = & \frac{a_{3}}{a_{1}}-\frac{3a_{2}^{2}}{8a_{1}^{2}};\;q=\frac{a_{2}^{3}-4a_{1}a_{2}a_{3}+8a_{1}^{2}a_{4}}{8a_{1}^{3}};\;S=\sqrt[{3}]{\frac{D_{1}+\sqrt{D_{1}^{2}-4D_{0}^{3}}}{2}}, \end{array}$$ (19)

$$\begin{array}{ccc} D_{0} & = & a_{3}^{3}-3a_{2}a_{4}+12a_{1}a_{5};\;D_{1}=2a_{3}^{3}-9a_{2}a_{3}a_{4}+27a_{2}^{2}a_{5}+27a_{1}a_{4}^{2}-72a_{1}a_{3}a_{5}. \end{array}$$ (20)

Since *a*_1_ < 0 and *a*_5_ > 0, the left hand side of (14) changes sign from positive at *α* = 0 to negative at *α* = *r* for all sufficiently large *r* > 0. So, (14) has at least one positive root.

Table 1 shows some of the important past studies conducted in the literature which can be deduced from the present formulation. The present formulation is important in the sense that it provides a wide range of parameter space for *γ*, *M*, *γ*_1_, *γ*_2_, and *V*_*c*_ in order to better analyze the underlying nonlinear boundary layer flow. Moreover, the closed form nature of the similarity solution is retained with the present more general formulation.

## 3 Numerical results and discussion

All numerical computations have been done in MATLAB programming. Since the roots of the polynomial Eq (14) have been found in closed form as in the preceding section, as such no numerical method is needed to further analyze the flow given by (13) and its dependency upon the various dimensionless parameters. The numerical results of our formulation are validated by reproducing the results of Bhattacharya et al. [23] by taking *γ*_1_ → 0, *γ*_2_ → 0, and *M* = 0 and that of Wu [36] by setting *M* = 0 and *γ* → ∞ in the present formulation. The numerical results are presented separately for shrinking and stretching sheet cases as follows.

### 3.1 Shrinking sheet case

**Fig 2:**
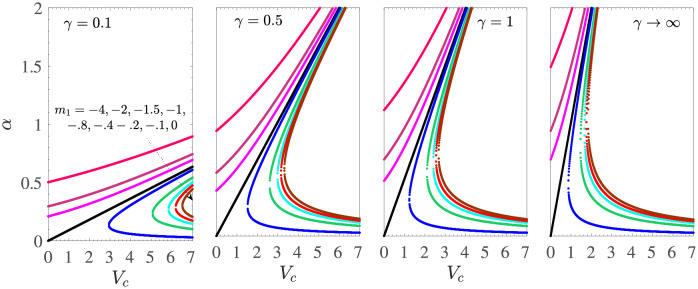
*α* vs *V*_*c*_ for *M* = 0, *γ*_1_ = 0.1 = −*γ*_2_, *d* = −(1 + *m*_1_) for the shrinking sheet case with mass suction.

Fig 2 shows the solution space in the (*α*, *V*_*c*_)-plane for various values of *m*_1_ and Casson parameter *γ* when the boundary layer flow is driven by the shrinking sheet with mass suction at the sheet surface. The fixed parametric values taken here are *γ*_1_ = 0.1 = −*γ*_2_, *d* = −(1+ *m*_1_), and *M* = 0. Different curves in each subfigure correspond to different values of *m*_1_. We first explain the curve *γ* = 0.1, where the Casson effects are prominent. The mass suction induced slip effect is strengthened on increasing |*m*_1_|. For *m*_1_ = −1 and *M* ≥ 0, we have the unique non-negative solution given by

$$\begin{array}{ccc} \alpha & = & \frac{\gamma }{1+\gamma }V_{c}, \end{array}$$ (21)

which is the threshold for two types of the solution regions described as follows:

(i) the unique solution region for *m*_1_ ≤ −1 which corresponds to $\alpha \geq \frac{\gamma }{1+\gamma }V_{c}$,
(ii) the dual solution region for *m*_1_ > −1 which corresponds to $\alpha <\frac{\gamma }{1+\gamma }V_{c}$.

Thus, as the Casson effects decrease, that is, from *γ* = 0.1 to *γ* → ∞, the region of the unique solution shifts upward, while the range of the dual solutions shifts to the left in the plane (*α*, *V*_*c*_). So, in the absence of the application of a magnetic field, the nature of the solution of the investigated MHD Casson model for the shrinking sheet depends on *γ*.

**Fig 3:**
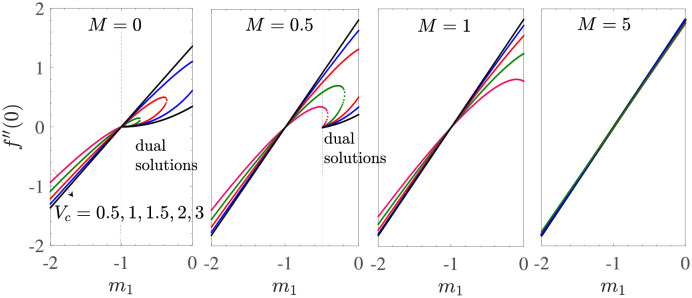
*f*′′(0) vs *m*_1_ for *γ* → ∞, *γ*_1_ = 0.1 = −*γ*_2_, *d* = −(1 + *m*_1_) for the shrinking sheet case with mass suction.

To see the dependence of the flow field in the vicinity of the shrinking sheet surface, we have obtained Fig 3 which shows the variation of the wall shear stress *f*′′(0) as a function of *m*_1_ for *γ* → ∞, *γ*_1_ = 0.1 = −*γ*_2_, *d* = −(1+ *m*_1_), −2 ≤ *m*_1_ ≤ 0 with mass suction. The different subfigures correspond to different values of *M*.

At the fixed value of the mass transfer parameter *V*_*c*_ and in the absence of magnetic effects (*M* = 0), the shear stress at the wall is an increasing function of *m*_1_ for −2 ≤ *m*_1_ < 0, the change is almost linear and *f*′′(0)<0. This shows that an increase in the amount of slip caused by mass suction results in a decrease in the shear stress at the wall in the shrinking sheet case. For *m*_1_ = −1, *f*′′(0) = 0 and *m*_1_ > −1, i.e. in the dual solution region, the two upper and lower branches of the solution *f*′′(0) remain positive and forms a closed curve in the region [−1, ∞)×[0, ∞) for (*m*_1_, *f*′′(0))-space when *M* = 0. The closed loop will open at *m*_1_ = −1 and will be larger, where the size of the loop is proportional to *V*_*c*_.

For *M* > 0, the unique solution region expands and the dual solution region narrows down in the (*f*′′(0), *m*_1_)-plane. This can be seen from the subfigure for *M* = 0.5 of Fig 3, where for a fixed value of *V*_*c*_, *f*′′(0) is an increasing function of *m*_1_ for −2 ≤ *m*_1_ < −0.45 with *f*′′(0) = 0 at *m*_1_ = −1. Here, dual solutions occur for *m*_1_ ≥ −0.50 and *f*′′(0) = 0 for *m*_1_ = −1 for all considered values of *V*_*c*_. The dual solution region does not occur for a sufficiently larger value of *M*, that is, *M* = 1. For *M* = 5, the variation of *f*′′(0) with *m*_1_ is not further affected by the suction. These observations show that in the absence of Casson effects, an increase in the applied magnetic field in the flow due to shrinking sheet would result in a unique flow field structure that is least dependent on the mass suction at the sheet wall. This is due to the presence of Lorentz force which results from electromagnetic interactions during the motion of the electrically conducting fluid. As *M* increases, the Lorentz force increases and resists flow. This stabilizing effect of the Lorentz force on the flow results in a reduction in the mass suction requirement. Thus, in sufficiently large magnetic fields with increased Lorentz force, the flow becomes independent of mass suction and eliminates the uncertainty of the flow dynamics. This makes the similarity solution unique.

**Fig 4:**
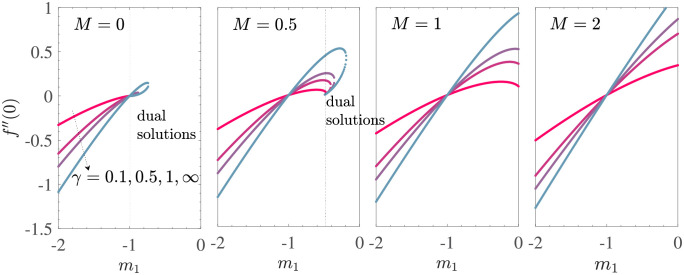
*f*′′(0) vs *m*_1_ for *V*_*c*_ = 1, *γ*_1_ = 0.1 = −*γ*_2_, *d* = −(1 + *m*_1_) for the shrinking sheet case with mass suction.

To observe the combined effect of the Casson parameter and the applied magnetic field on the flow field, we present Fig 4 which shows that variation of wall shear stress *f*′′(0) with *m*_1_ for a set of values of *γ* and *M*. The fixed parametric values are *V*_*c*_ = 1, *γ*_1_ = 0.1 = −*γ*_2_, and *d* = −(1+ *m*_1_).

**Fig 5:**
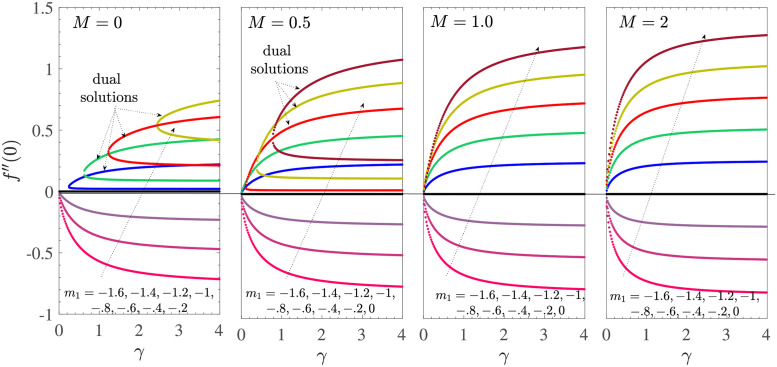
*f*′′(0) vs *γ* for *V*_*c*_ = 2, *γ*_1_ = 0.1 = −*γ*_2_, *d* = −(1+ *m*_1_) for the shrinking sheet case with mass suction.

Here, the nature of the changes in *f*′′(0) and *m*_1_ is analogous to that shown in Fig 5. As the *M* increase, the region of unique solutions expands, as before. Thus, we concluded that the effect of the applied magnetic field on the MHD flow studied is similar in the absence and presence of Casson effects.

To understand the dependence of the wall shear stress due to the boundary layer flow of the MHD Casson fluid on *γ*, we obtained Fig 5 which shows the plots of *f*′′(0) with the Casson parameter *γ* for mass suction at the sheet wall with the fixed parameter values of *V*_*c*_ = 2, *γ*_1_ = 0.1 = −*γ*_2_, and *d* = −(1+ *m*_1_). The curves of each subfigure are shown for different values of the slip parameter *m*_1_ induced by mass suction. For the unique solution region (*m*_1_ ≤ −1), for a given value of *m*_1_ and any value of the magnetic parameter *M*, *f*′′(0)≤0 for all *γ*. In addition, the wall shear stress *f*′′(0) decreases rapidly with *γ* for 0 < *γ* < 1, where the variation is negligible for *γ* > 3. This shows an enhancement in the magnitude of the wall shear stress due to addition of the yield stress of the Casson fluid. The correlation of the four subfigures in Fig 5 depicts that an increase in the applied magnetic field as well as in |*m*_1_| further increases the wall shear stress due to the flow driven by the shrinking sheet. On the other hand, behavior of the variation of *f*′′(0) with *γ* is different in the dual solution region (*m*_1_ > −1). Here *f*′′(0)>0 for all considered parametric values. For *M* = 0, *γ* has a minimum value of *γ*_*s*_ > 0 (depending on *m*_1_), so there is a dual solution for all *γ* ≥ *γ*_*s*_. At *γ* = *γ*_*s*_, the solution bifurcates into upper and lower branch, where the solutions represented by the upper and the lower branches are increasing and decreasing functions of *γ*. There are a total of 4 dual solutions in the region 0 < *γ* ≤ 4 for *M* = 0. Considering MHD effects, the subfigures *M* = 0.5 show that an increase in *M* results in an increase in the gap between the upper and lower branches of dual solutions. The lower solution branch tends to flatten out and eventually disappears if it reaches a large enough value of *M*.

A closer comparison of all 4 subsets shows that *γ*_*s*_ decreases with increasing *M* and *γ*_*s*_→ 0 for each sufficiently large *M* value when there is no more region of the dual solutions. This again confirms that the increased MHD effects tend to broaden the range of unique solutions in the present Casson model for the shrinking sheet with suction.

**Fig 6:**
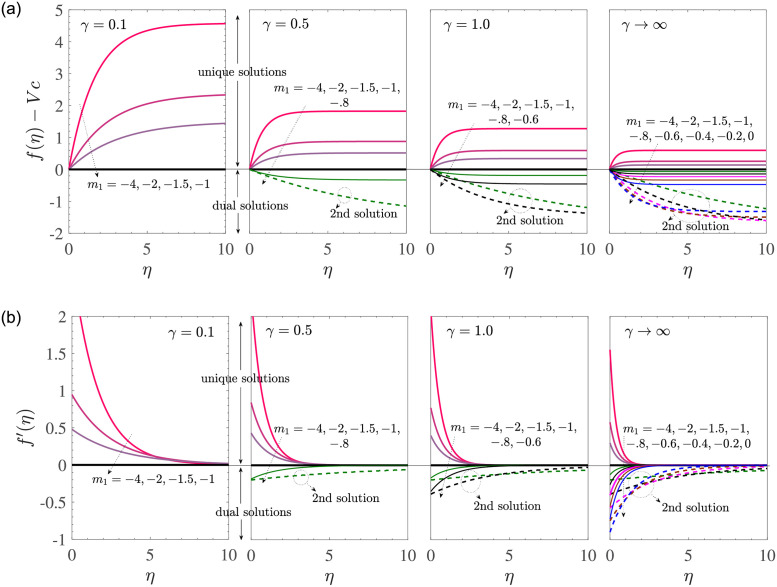
*f*(*η*)−*V*_*c*_ and *f*′(*η*) vs *η* for *M* = 0, *V*_*c*_ = 2, *γ*_1_ = 0.1 = −*γ*_2_, *d* = −(1 + *m*_1_).

Fig 6 depicts the variations of the two velocity profiles *f*(*η*)−*V*_*c*_ and *f*′(*η*) with *η* for *M* = 0, *V*_*c*_ = 2, *γ*_1_ = 0.1 = −*γ*_2_, *d* = −(1+ *m*_1_) for the MHD Casson fluid flow due to a shrinking sheet with mass suction. The curves in each subfigure were made for distinct values of *m*_1_. For *γ* = 0.1 when the Casson effects are dominant, *f*(*η*) is an increasing function of *η* and *f*′(*η*) is a decreasing function of *η*. Here, unique solutions exist since 0.1 < *γ*_*s*_. For *γ* = 0.5, a pair of dual solutions exists for *m*_1_ = −0.8; each solution for *f*(*η*) decreases with *η* and each of the dual solutions for *f*′(*η*) increases with *η*. An additional increase in *γ* (decrease in Casson effects) results in several dual solutions. However, increasing *γ* decreases the values of *f*(*η*) and *f*′(*η*) at a given point in the boundary layer. In the presence of MHD effects (*M* ≠ 0), the Cas-son effects are amplified and the corresponding calculations are omitted.

**Fig 7:**
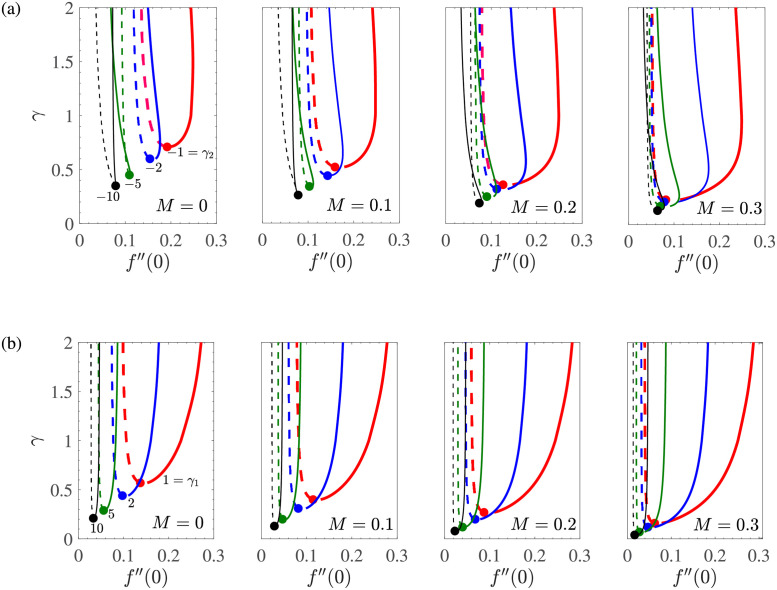
*γ* vs *f*′′(0) for *V*_*c*_ = 2 and *m*_1_ = −0.5 for the shrinking sheet case with mass suction. All the cases considered here correspond to the dual solutions. The pair of (one dashed and one solid curve) solutions in each case is separated by the bifurcation point •.

It is not yet clear how the Navier’s slip parameter *γ*_1_ and the second order slip parameter *γ*_2_ affect the boundary layer flow. In this regards, we have obtained Fig 7 which shows the variation of Casson parameter *γ* with the wall shear stress *f*′′(0) for *V*_*c*_ = 2, *m*_1_ = −0.5, and *M* = 0, 0.1, 0.2, 0.3. These parameter values correspond to the dual solution regions, and here, we are interested in the effects of the two types of slip parameters on the dual solutions. In Fig 7a, we have taken *γ*_1_ = 0.01 to observe the effect of change of *γ*_2_ on the wall shear stress. For a fixed value of *M*, with an increase in the value of |*γ*_2_|, *f*′′(0) occurs at a comparatively low value, which shows that the wall shears stress decreases for all sufficiently large Casson effects when the second order slip effects are increased. An increase of |*γ*_2_| also results in decrease of the gap between the two solution branches.

The dependence of *f*′′(0) on the Navier’s slip parameter is similar, as shown in Fig 7b, in which *γ*_2_ = −0.01 is taken. The role of the applied magnetic field is to widen the gap between the double solutions in each case.

It can be observed from the present formulation and numerical computation that there is no stable solution in the case when the flow is driven by a shrinking sheet with mass injection with or without mass injection induced slip.

### 3.2 Stretching sheet case

**Fig 8:**
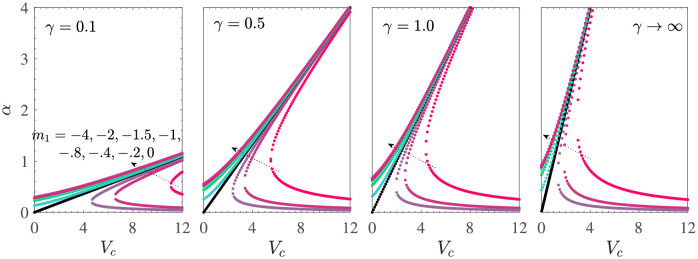
*α* vs *V*_*c*_ for *γ*_1_ = 0.1 = −*γ*_2_ and *d* = 1 + *m*_1_ for the stretching sheet case with mass suction. Here, unique solutions occur for *m*_1_ ≤ −1−*M* and the dual solutions occur for *m*_1_ > −1−*M*.

The location of the root *α* of (14) with respect to *V*_*c*_ for various values of *m*_1_ (here *d* = 1 + *m*_1_) is analogous to the case of shrinking sheet as shown in Fig 8. Here also, the plane (*α*, *V*_*c*_) is divided into the unique solution region for *m*_1_ ≥ −(1+ *M*) and the dual solution region for *m*_1_ < −(1+ *M*). These regions are separated by the straight line corresponding to *m*_1_ = −1−*M*. Here, the effect of increasing *M* remains similar to that of the earlier case of shrinking sheet. In fact, all the other numerical inferences on stretching sheet case with mass suction are also analogous to that of the shrinking sheet case with mass suction, and we skip the corresponding calculations.

**Fig 9:**
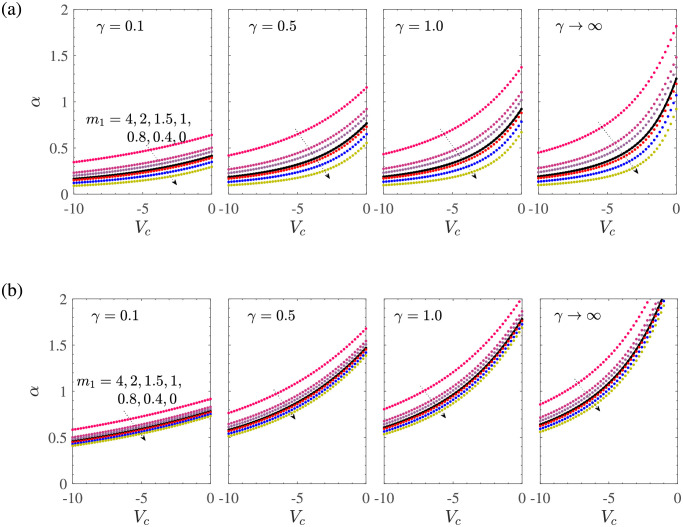
*α* vs *V*_*c*_ for *γ*_1_ = 0.1 = −*γ*_2_ and *d* = 1 + *m*_1_ for the stretching sheet case with mass suction. Here, unique solutions occur for *m*_1_ ≤ −1−*M* and the dual solutions occur for *m*_1_ > −1−*M*.

Here we emphasize the case of stretching sheet with mass injection. Wu [36] has found that for the Newtonian fluid (*γ* → ∞), the boundary layer flow due to stretching sheet with mass injection and the first and second order slip is unique for all permissible values of the mass transfer parameter *V*_*c*_ and *m*_1_. The uniqueness holds in the more general case of MHD Casson fluid flow as can be seen from Fig 9a and 9b, which show the variation of *α* with *V*_*c*_ for *γ*_1_ = 0.1 = −*γ*_2_ and *d* = 1+ *m*_1_. Apart from the uniqueness of the solution, we observe from Fig 9a that for *M* = 0 and *γ* = 0.1, all the solutions lie in the small interval 0 < *α* ≤ 0.6 when *V*_*c*_ is varied from −10 to 0. The solution *α* is an increasing function of *V*_*c*_ as well as *m*_1_ for all values of *γ*, where the rate of increase is large in a neighborhood of *V*_*c*_ = 0. An increase in *γ* results in increase of the size of the interval of the solution *α*. In the presence of MHD effects, that is, *M* = 5, we can infer from Fig 9b that the value of *α* corresponding to a given value of *m*_1_ and *V*_*c*_ occurs at a larger value than the one occurring for *M* = 0 for all values of *γ*. However, an increased value of *M* also results in contraction of the size of the interval of the solution *α*. Since

$$\begin{array}{ccc} f^{\prime \prime }\left(0\right) & = & \frac{-\alpha d}{1+\gamma_{1}\alpha -\gamma_{2}\alpha^{2}}, \end{array}$$ (22)

and for the flow due to stretching sheet with injection, *d* = 1 + *m*_1_ with *m*_1_ ≥ 0, then it can be deduced that *f*′′(0) is a decreasing function of *α* for all *α* satisfying $\alpha^{2}>\frac{1}{-\gamma_{2}}$ and *f*′′(0) is an increasing function of *α* for $\alpha^{2}<\frac{1}{-\gamma_{2}}$. In particular, for −*γ*_2_ = 0.1, *f*′′(0) is decreasing with *α* for all $\alpha >\sqrt{10}$ and increasing for $0<\alpha <\sqrt{10}$. Also, from Fig 9 we see that for *γ* = 0.1, we have $0<\alpha <1<\sqrt{10}$ and a decrease in the fluid injection at the sheet wall results in an increased value of *α* and hence of *f*′′(0). Thus, for *γ*_2_ = −0.1 and when the Casson effects are dominant, that is, *γ* = 0.1, the wall shear stress will increase on reducing the mass injection at the stretching sheet in absence as well as in presence of the magnetic field.

**Fig 10:**
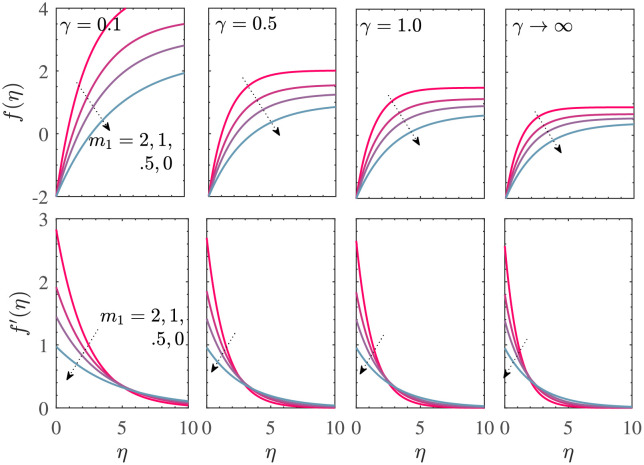
*α* vs *V*_*c*_ (a) *M* = 0 (b) *M* = 5. The fixed parametric values are *γ*_1_ = 0.1 = −*γ*_2_, *d* = 1+ *m*_1_ for the stretching sheet case with mass injection. Here, all solutions are found to be unique.

The corresponding velocity profiles are shown in Fig 10 for *M* = 0, *V*_*c*_ = −2, *γ*_1_ = 0.1 = −*γ*_2_, *d* = 1+ *m*_1_. For a given value of *γ*, the boundary layer thickness decreases with the increase in *m*_1_. The Casson fluid flow has a larger vertical velocity at a particular location as compared to that in the case of Newtonian fluid for each value of *m*_1_. Also, the vertical velocity is positive due to mass injection in the region near the sheet but the vertical velocity eventually becomes negative for sufficiently large distance from the sheet. For a fixed value of *m*_1_, an increase in the Casson parameter *γ* results in significant decrease of the vertical velocity at any given location *η* in the boundary layer. The vertical velocity *f*′(*η*) is positive and is a decreasing function of *η* for all values of *m*_1_ and *γ*. The profiles for *M* > 0 are similar to those obtained here for *M* = 0 and we skip the corresponding description.

The present work was motivated by medical situations in hemodynamics, where medicine-mixed MHD particles are injected into the bloodstream close to the tumor site, while subjecting the tumor area to an external magnetic field. These particles act like a heat source in the presence of a magnetic field causing the cancer cells of the tumor tissue get destroyed. The analogy of hemodynamics with the presently considered flow problem can be understood as follows: in such a cardiovascular system, blood is a typical Casson fluid and the inner surface of blood vessels under a controlled magnetic field can be modeled as a stretching sheet problem, where the wall of the blood vessel behaves like a stretching sheet [44]. In view of the present work, the wall shear stress at the inner wall of the blood vessels is likely to play significant role in the dynamics of blood flow.

## 4 Conclusions

In the present study, the complex boundary layer flow of a Casson fluid under MHD conditions due to a stretching/shrinking sheet with mass suction/injection is considered. The modified Navier’s slip and second order slip conditions due to mass suction/injection are also imposed on the underlying boundary layer flow of the Casson fluid. The flow characteristics have been expressed in terms of the Casson parameter *γ* and the magnetic parameter *M*.The numerical results are obtained for the shrinking as well as the stretching sheet cases separately. At the end of this study, we conclude the following results can be discussed as follows

- For the shrinking sheet case with mass suction at the sheet surface when the Casson effects are dominant, the mass suction induced slip effect gets strengthened on increasing the parameter |*m*_1_|.
- The interval of solution for α becomes small under the Casson effects in comparison to the Newtonian fluid flow considerations. The solution space in (*V*_*c*_, *α*)-plane consists of unique solutions for *m*_1_ ≤ −1 and dual solutions for *m*_1_ > −1.
- An increase of the magnetic effects results in expansion of the unique solution region and contraction of the dual solution region for all considered parametric values under mass suction. This occurs due to the action of Lorentz force induced in the flow as a result of the interaction between the electric field and magnetic field within the flow.
- In the unique solution region, an increase in the magnitude of mass suction induced slip results in the reduction of the wall shear stress in the shrinking sheet. On the other hand, an increase in Casson effects, the applied magnetic field and |*m*_1_| result in an enhancement of the magnitude of the wall shear stress due to the flow induced by the shrinking sheet with mass suction.
- In the dual solution region, the dependence of the wall shear stress due to shrinking sheet with mass suction on the Casson effects is different from the one that exists for the case of a unique solution region. The Casson effects further get amplified in the presence of a magnetic field.
- An increase of the magnitude of the first/second order slip parameters results in a decrease of the wall shear stress at the shrinking sheet surface and also in a decrease of the gap between the dual solution branches.
- For the stretching sheet case with mass suction, the location of the solution and the effect of the applied magnetic field remains similar to that existing for the shrinking sheet case.

## Glossary

*Subscripts*

*A* and *B*: Coefficients of 1st and 2nd order slips respectively
D: Component of deformation rate
d: Parameter of proportional shearing
*D*_*c*_: Critical value
*f*(*η*): Dimensionless stream function
*H*_0_: Vertical magnetic field (T)
L: Characteristic scales for measuring horizontal length (m)
*U*_0_: Characteristic scales for measuring horizontal velocity
*V*_0_: Suction/injection velocity (ms^−1^)
*V*_*c*_: Suction/injection parameter
(*x*, *y*): Cartesian coordinates (m)

*Greek symbols*

α: Solution domain
γ: Casson fluid parameter
*γ*_1_ and *γ*_2_: First and second order slip parameter ()
δ: Thickness of the laminar boundary
η: Similarity variable
*μ*_*B*_: Active viscosity of the non-Newtonian liquid (Pa.s)
ν: Kinematic viscosity (m 2s -1)
ρ: Density of the base fluid (kg.m -3)
σ: Electrical conductivity (S.m -1)
*τ*_*y*_: Yield stress (N.m -2)
ψ: Stream function

*Subscripts*

*w*: Quantities at the wall
∞: Quantities at the free stream
